# TP53 codon 72 polymorphism affects accumulation of mtDNA damage in human cells

**DOI:** 10.18632/aging.100425

**Published:** 2012-01-29

**Authors:** Serena Altilia, Aurelia Santoro, Davide Malagoli, Catia Lanzarini, Josué Adolfo Ballesteros Álvarez, Gianluca Galazzo, Donald Carl Porter, Paolina Crocco, Giuseppina Rose, Giuseppe Passarino, Igor Boris Roninson, Claudio Franceschi, Stefano Salvioli

**Affiliations:** ^1^ Department of Experimental Pathology, University of Bologna, Bologna, 40126, Italy; ^2^ Ordway Research Institute, Albany NY 12208, USA; ^3^ Interdepartmental Centre “L. Galvani” (C.I.G.), University of Bologna, Bologna, 40126, Italy; ^4^ Department of Animal Biology, University of Modena and Reggio Emilia, Modena, 41100, Italy; ^5^ Senex Biotechnology, Inc., CLS Building, Columbia SC 29208, USA; ^6^ Department of Cell Biology, University of Calabria, Rende, 87030, Italy; ^7^ Department of Pharmaceutical and Biomedical Sciences, University of South Carolina, Columbia SC 29208

**Keywords:** p53 codon 72 polymorphism, mitochondrial DNA, polymerase gamma, aging, mtDNA heteroplasmy

## Abstract

Human TP53 gene is characterised by a polymorphism at codon 72 leading to an Arginine-to-Proline (R/P) substitution. The two resulting p53 isoforms have a different subcellular localisation after stress (more nuclear or more mitochondrial for the P or R isoform, respectively). p53P72 variant is more efficient than p53R72 in inducing the expression of genes involved in nuclear DNA repair. Since p53 is involved also in mitochondrial DNA (mtDNA) maintenance, we wondered whether these p53 isoforms are associated with different accumulation of mtDNA damage. We observed that cells bearing p53R72 accumulate lower amount of mtDNA damage upon rotenone stress with respect to cells bearing p53P72, and that p53R72 co-localises with polymerase gamma more than p53P72. We also analysed the in vivo accumulation of heteroplasmy in a 300 bp fragment of mtDNA D-loop of 425 aged subjects. We observed that subjects with heteroplasmy higher than 5% are significantly less than expected in the p53R72/R72 group. On the whole, these data suggest that the polymorphism of TP53 at codon 72 affects the accumulation of mtDNA mutations, likely through the different ability of the two p53 isoforms to bind to polymerase gamma, and may contribute to in vivo accumulation of mtDNA mutations.

## INTRODUCTION

Mitochondrial DNA (mtDNA) is a small, double-stranded circular molecule of 16,569 bp that is contained in many copies inside mitochondria, packaged into multimeric complexes called nucleoids made up of proteins and nucleic acids [1]. MtDNA molecules are particularly exposed to the reactive oxygen species (ROS) that are formed by the incomeplete oxygen reduction due to electron leakage from the transport chain and therefore are very susceptible to oxidative damage as well as to other mutagenic lesions, also because of a limited capability of their repair systems [2].

A key molecule for mtDNA stability is polymerase gamma (polg), which is the sole mitochondrial polymerase and plays an essential role in both replication and repair of the mtDNA [3,4]. Human polg is a heterotrimer that contains a 140 kDa catalytic subunit and a homodimer of accessory subunits. A polg defective in proofreading activity has been associated to a phenotype of accelerated aging in a murine model [5,6]. Alterations in the polymerase and exonuclease activity of polg induce an increase in the frequency of mtDNA mutations [3,7] and mutations of POLG1 gene are associated with a number of diseases [8-10]. Data also suggest that polg is likely involved in the aging process [11] and in the pathogenesis of a series of diseases in which mtDNA instability plays a pathogenic role, such as age-associated sarcopenia and Parkinson's Disease [12,13]. Nevertheless, the molecular details by which polg ensures mtDNA integrity are still to be elucidated.

It has been reported that p53 plays a role in the maintenance of mtDNA integrity [14-17]. TP53 gene has a well-known crucial role in the response to DNA damage, in cell cycle regulation, apoptosis and cell senescence. Recent studies from our and other laboratories demonstrated that p53 protein, the product of TP53 gene, in response to an oxidative stress not only behaves as a transcription factor at nuclear level, but can also translocate at mitochondrial level [18-20]. In the mitochondria, p53 could bind to Bcl-xL and to other proteins to induce mitochondrial-mediated apoptosis independently from transcriptional activity [18-21]. Moreover, it has been observed that a fraction of p53 is present in mitochondria of non-stressed cells [21]. It has been reported that within mitochondria p53 can bind to polg and modulate its activity [14,15]. Indeed, it has been observed that in TP53 knock out animals the base excision repair activity is less efficient, and that p53 can bind to polg in *in vitro* cellular models, increasing polg-mediated mtDNA replication and suppressing the mutagenic effect of ROS and ethidium bromide [14,15,22]. It is also reported that in mtDNA at least one consensus sequence for p53 binding does exist [23].

TP53 gene has a number of natural allelic variants, among which those due to the polymorphism at codon 72 (in the exon 4) are of particular interest. This common polymorphism causes a C-to-G transversion that in turn leads to a Proline-to-Arginine substitution in the p53 protein. The two resulting variants (p53P^72^ and p53R^72^) are different as far as the capability to modulate apoptosis, to translocate to mitochondria, to be degraded by proteasome and to bind to MDM2 [24-27]. It has been observed that these differences become significant in *ex vivo* models as the age of the donor increases, being negligible in cells from young donors, and statistically significant in cells from old people and centenarians [20,28]. The *in vivo* functional importance of such a polymorphism is demonstrated by the fact that p53R^72^ homozygotes and p53P^72^ carrier subjects have a different survival after age 85 (greater for p53P^72^ carriers) as well as a different cancer incidence and survival after cancer diagnosis [29-31].

It has been reported that p53P^72^ is more able than p53R^72^ in promoting nuclear DNA repair [32]. Since as summarised above the two p53 isoforms have a different tendency to localise at mitochondria, we then wondered whether they can differ also in capability to maintain mtDNA stability, and whether this may occur through a differential binding to mtDNA replisome components such as polg. To check this hypothesis we performed *in vitro* as well as *in vivo* studies whose results suggest that this is the case.

## RESULTS

### p53R^72^ localises with extranuclear 8-oxo-dG more than p53P^72^ and protects mitochondrial function

**Figure 1:**
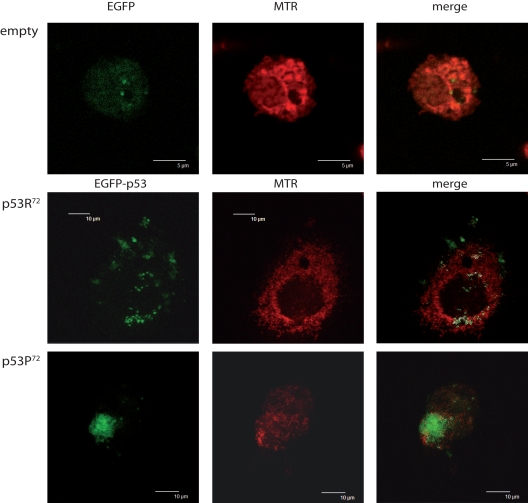
Different localisation of p53 isoforms after rotenone treatment. p53^−/−^ HCT116 cells transfected with empty EGFP pCMS plasmid (upper panels), EGFP-p53R^72^ pCMS plasmid (central panels), or EGFP-p53P^72^ pCMS plasmid (lower panels) and counterstained with MitoTracker Red (MTR). Cells transfected with the empty vector show a diffused EGFP fluorescence, not associated with any cell structure; in cells transfected with the plasmids expressing the p53 isoforms, a different subcellular localisation is noticed, as assessed by counter-staining with the mitochondrial-specific probe MTR. In particular, when EGFP-p53R^72^ pCMS plasmid is used, EGFP fluorescence, associated to p53 protein, appears to be not nuclear but rather co-localised with MTR fluorescence, indicating that p53 has a mitochondrial localisation (white dots indicate points of overlapping of the two fluorescences).

We could confirm that, as previously reported for *ex vivo* cells [20], also ectopically expressed p53 tends to localise differently according to the polymorphism at codon 72. Figure 1 shows the confocal analysis of p53 null HCT116 cells transfected with pCMS-EGFP plasmid, either empty or expressing the arginine or proline TP53 allele. Three representative cells are presented. It can be appreciated that upon treatment with 100 nM rotenone (an inhibitor of mitochondrial respiration complex I) for 24 hours, while cells transfected with the empty vector resulted to have a widespread EGFP fluorescence not clearly associated with any subcellular structure (upper panels), cells transfected with p53R^72^-expressing plasmid showed a dotted EGFP fluorescence largely overlapping with MitoTracker Red CMX-Ros (MTR) fluorescence specific for mitochondria (central panels), a phenomenon which is much less evident when p53P^72^-expressing plasmid is used (lower panels).

**Figure 2:**
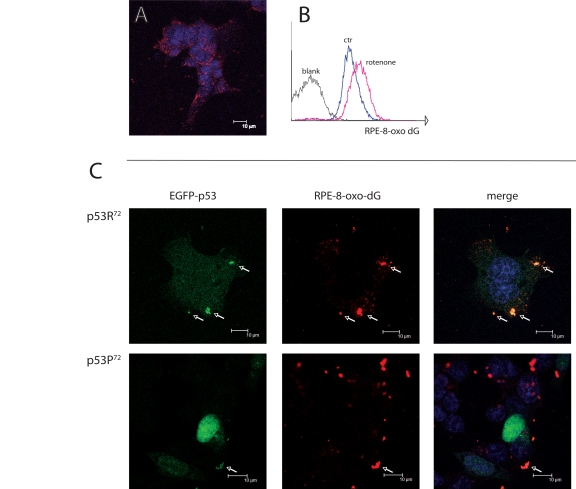
Co-localisation of p53 isoforms with damaged mtDNA. (**A**) p53^−/−^ HCT116 cells treated with 100 nM rotenone for 24 hours and stained for 8-oxo-dG and revealed with RPE-conjugated secondary moAb (red fluorescence). Nuclei are counterstained with Hoechst 33258. Punctuated, cytoplasmic red fluorescence indicates that 8-oxo-dG accumulates in mitochondria but not nuclei upon rotenone treatment. (**B**) flow cytometric detection of 8-oxo-dG after rotenone treatment. **C**: p53^−/−^ HCT116 cells transfected with either EGFP-p53R^72^ or EGFP-p53P^72^ pCMS plasmid and treated as in **A**. Arrows indicate the points in which 8-oxo-dG (red fluorescence) and p53 (green fluorescence) co-localise (yellow dots in the merged picture). Note that in the case of p53R^72^ the green fluorescence is diffused in the cytoplasm and tends to accumulate in mitochondria, while in the case of p53P^72^ the green fluorescence is mainly localised to the nucleus.

Upon treatment with rotenone, we observed by confocal microscopy and flow cytometry (Figure 2A and 2B) a consistent accumulation of 8-oxo-dG fluorescence, which appears to be localised outside the nucleus of cells (Figure 2A). When comparing cells transfected with either EGFP-p53R^72^ or EGFP-p53P^72^ expressing plasmids, we observed that upon rotenone treatment p53R^72^ tends to co-localise with 8-oxo-dG extranuclear fluorescence more than p53P^72^ (Figure 2C, arrows). On the other side, p53P^72^ has a preferential nuclear localisation, as showed by the perfect overlap with Hoechst staining. This indicates that rotenone treatment induces formation of 8-oxo-dG adducts in mitochondria, the target of rotenone and the sole organelle bearing extranuclear DNA, and that p53R^72^ isoform tends to co-localise with these adducts.

**Figure 3:**
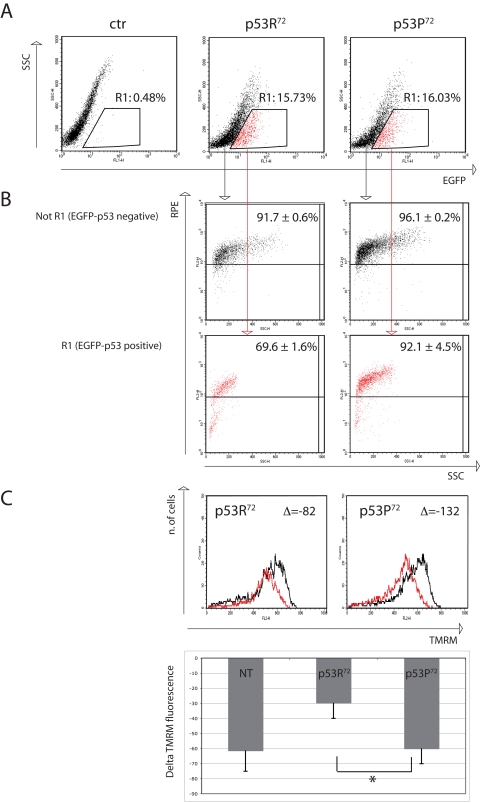
Flow cytometry analysis of p53^−/−^ HCT116 cells transfected with either EGFP-p53R^72^ or EGFP-p53P^72^ pCMS plasmid. After 24 hours from transfection, cells were treated with 100 nM rotenone and incubated for additional 24 hours, then stained for 8-oxo-dG or TMRM (see materials and methods). (**A**) transfection efficiency. Cells contained in R1 are considered EGFP-positive. Ctr: non-transfected cells. (**B**) 8-oxo-dG detection. The cells in (R1) and (not R1) are evaluated for 8-oxo-dG fluorescence (RPE). Numbers represent the percentage of cells with high 8-oxo-dG fluorescence and are expressed as mean ± st. dev. of three independent experiments. As showed EGFP-negative cells (not R1, not expressing p53) are almost all positive for 8-oxo-dG fluorescence (more than 90%), while when considering EGFP-positive cells (R1, expressing p53), the cells that were transfected with p53R^72^ which resulted positive for 8-oxo-dG decreased to 69%, with respect to 92% of those that were transfected with p53P^72^. See text for comment. (**C**) Mitochondrial membrane potential (MMP) analysis. The cells in (R1) were evaluated for MMP by using the potentiometric dye TMRM. Black line: control cells; red line: rotenone treatment. Decrease in MMP was expressed as the difference (Δ) of TMRM fluorescence intensity between rotenone-treated and control cells. Cells positive for EGFP-p53R^72^ (left panel) display a lower decrease in MMP with respect to those positive for EGFP-p53P^72^ (right panel). The graphic shows data related to 3 independent experiments (mean ± st. dev.) * Student' *t* test p= 0.022. NT, not transfected.

In order to have a semi-quantitative evaluation of the accumulation of 8-oxo-dG in cells transfected with either EGFP-p53R^72^ or EGFP-p53P^72^ we set up a flow cytometric test in which transfected HCT116 cells were treated with rotenone 100nM for 24 hours and then immunostained with anti-8-oxo-dG moAb. Cells positive for EGFP (expressing p53) were electronically gated and compared for 8-oxo-dG specific fluorescence (RPE fluorochrome) with EGFP-negative cells (not expressing p53). Results are reported in Figure 3, where a representative experiment is reported together with mean ± st.dev. of three separate experiments. Figure 3A shows the transfection efficiency, which is about 16% for both plasmids. Cells considered EGFP-positive are gated in Region 1 (R1) and represented in red dots. Figure 3B shows the 8-oxo-dG fluorescence of cells not gated in R1 (upper panels) and gated within R1 (lower panels). As shown, EGFP-p53-negative cells (not R1) are almost all positive for 8-oxo-dG fluorescence (more than 90%), no matter of the plasmid used for the transfection procedure (EGFP-p53R^72^ pCMS *vs* EGFP-p53P^72^ pCMS, p=0.34, two-tailed Student' *t* test). On the contrary, when considering EGFP-p53-positive cells (R1), the percentage of cells positive for 8-oxo-dG is quite different depending on the p53 isoform the cells are transfected with. Indeed, this percentage is consistently lower for cells expressing the p53R^72^ isoform with respect to those expressing the p53P^72^ one (69.6 ± 1.6 *vs* 92.1 ± 4.5, p=0.003). An analysis of Mitochondrial Membrane Potential (MMP) made with the potentiometric dye TMRM also indicated that EGFP-p53R^72^-positive cells are less susceptible to undergo MMP decrease with respect to those positive for EGFP-p53P^72^, suggesting that these cells have a better preserved mitochondrial function (Figure 3C).

### p53R72 binds to polg more than p53P72 and protects mtDNA integrity

**Figure 4:**
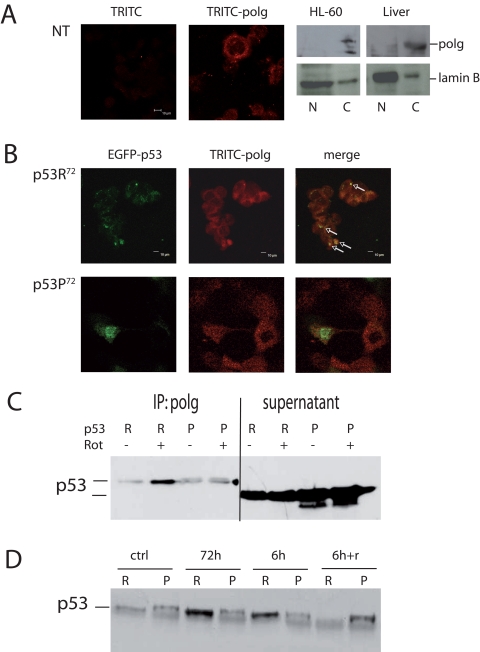
Co-localisation of p53 and polg. (**A**) Confocal analysis of p53^−/−^ HCT116 non-transfected cells stained with anti-polg antibody alone or with the TRITC-conjugated secondary antibody; Western Blot analysis of specificity of the anti-polg antibody on cytoplasmic and nuclear fractions obtained from HL-60 cells and human hepatocytes. As shown, the band corresponding to the molecular weight of polg is present only in the cytoplasmic fractions. (**B**) Confocal analysis of p53^−/−^ HCT116 cells transfected with EGFP-p53R^72^ (upper panels) or EGFP-p53P^72^ (lower panels) pCMS plasmids and treated with 100 nM rotenone for 24h. It is possible to observe that only in the case of p53R^72^ many points of co-localisation are visible (arrows). (**C, D)** Co-Immunoprecipitation assay on stably transfected PC3 cells. In C cells were treated for 72h with 10 nM rotenone. R= p53R^72^; P= p53P^72^. The lanes indicated as supernatant show the amount of p53 expressed by the cells. In D cells were exposed to 10 nM rotenone for 6h, 72h, 6h and recovery until 72h (6h+r).

A possible mechanism to explain this difference could be the reported different capability of the two p53 isoforms to induce apoptosis, so that heavily damaged cells transfected with p53R^72^ isoform would be more prone to apoptosis than those transfected with p53P^72^, but this seems not to be the case. Indeed, transfection appears to induce massive apoptosis, but with no difference between the two plasmids (data not shown). Another possible mechanism is the different capability of the two isoforms to localise at mitochondrial level. This may favour the binding and activation of polg, the polymerase involved in mtDNA repair. We then checked the co-localisation of p53 isoforms with polg. After checking for anti-polg antibody specificity (Figure 4A), we found that EGFP-p53R^72^ co-localises with polg more than EGFP-p53P^72^ (Figure 4B, arrows).

To further strengthen these data, we generated stably transfected PC3 cells expressing either p53R^72^ or p53P^72^, conjugated with HA epitope tag, and performed co-immunoprecipitation assay (Figure 4C). To this purpose, cells were exposed to10 nM rotenone, for 72 hours then protein were precipitated for polg and assayed with anti-HA antibody. These experiments confirmed that p53R^72^ is more bound to polg than p53P^72^. The experiment was repeated with different exposure times. Other than 72 hours exposure, cells were treated for 6 or for 6 hours then recovery until 72 hours (6h+r). Also in this case p53R^72^ resulted to be more bound to polg than p53P^72^ except that at point 6h+r (Figure 4D).

**Figure 5:**
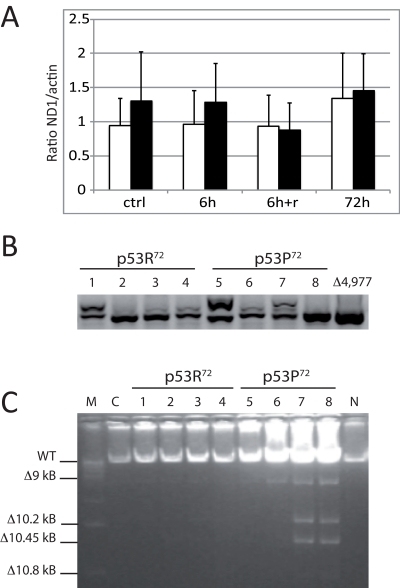
Measurements on mtDNA of stably transfected PC3 cells. Cells were exposed to 10 nM rotenone for 6h, 72h, and 6g and recovery until 72h (6h+r). (**A**) copy number assessed by Real-time PCR. Data are expressed as ratio between the levels of ND1 and actin genes, and are the average value of three separate measurements ± st. dev. White columns: p53R72 expressing cells; black columns: p53P72 expressing cells. (**B**) Nested PCR specific for the 4,977 bp deletion. A representative experiment out of three is showed. 1,5: control; 2,6: 6h; 3,7: 6h+r; 4,8: 72h; Δ4,977: DNA from a subject carrying the 4,977 bp deletion. **C**: Long PCR for multiple mtDNA deletions. Numbers legend as in B; M: marker 1 Kb; C: non-transfected PC3 cells; N: DNA from PBMC of a young subject.

It is reported that mutator mice with a polg deficient in proofreading activity accumulate both mtDNA point mutations and deletions [33]. We then checked whether exposure to rotenone in cells expressing either one or the other p53 isoform could induce an accumulation of mtDNA damage such as large deletions (*e.g.* the 4,977 bp), assessed by nested PCR and Long PCR. Also, since this deletion leads to an increase in mtDNA copy number [34], we also checked whether mtDNA copy number, assessed by Real-time PCR, is affected in stably transfected cells. These tests were performed on total DNA extracted from stably transfected PC3 cells before and after treatment with 10 nM rotenone. No difference in terms of mtDNA copy number was observed (Figure 5A), even if a trend of higher copy number is present for p53P^72^ expressing cells. Nevertheless, both Nested PCR and Long PCR indicated that these cells accumulate higher levels of deletions in the mtDNA as compared to those expressing p53R^72^ (Figure 5B,C).

As a whole, these data suggest that TP53 codon 72 polymorphism affects accumulation of damage in mtDNA likely by modulating the efficiency of mtDNA repair. Data presented in this paper suggest that this modulation occurs through the binding of p53 to polg, even if other possible mechanisms can not be excluded.

### TP53 codon 72 polymorphism is associated with mtDNA heteroplasmy *in vivo*

In order to investigate whether TP53 codon 72 polymorphism has an impact on mtDNA stability *in vivo*, thus reflecting the differences we observed for the two isoforms *in vitro*, we analysed the correlation between mtDNA heteroplasmy and TP53 codon 72 genotype in white blood cells (WBC) from 425 volunteers of old age (from 60 to 108 years of age), where accumulation of somatic mtDNA mutations are more likely to occur. Heteroplasmy was assessed by DHPLC in a segment of the mtDNA D-loop from nt 16531 to nt 261, spanning between hypervariable region (HVR) 1 and 2. Table 1 shows the distribution of mtDNA heteroplasmy in subjects with different genotypes at TP53 codon 72. We found that subjects with different level of heteroplasmy are not randomly distributed across the three genotypes (p=0.0147). In particular, considering a heteroplasmy threshold of 5%, we observed that homozygous TP53R^72^/R^72^ subjects with a heteroplasmy over 5% are less frequent than expected, while heterozygous subjects with a heteroplasmy over 5% are more frequent than expected. A univariate logistic analysis, where the genotype at TP53 codon 72 was the independent variable and the presence/absence of high level of heteroplasmy was the dependent variable, showed that p53R^72^ acts as a recessive allele on the trait presence/absence of high level of heteroplasmy. On the whole these data suggest that homozygous TP53R^72^/R^72^ genotype is associated with lower accumulation of heteroplasmy in the considered mtDNA region.

**Table 1:** Distribution of TP53 codon 72 genotypes across the two groups of subjects defined on the basis of the *in vivo* mtDNA heteroplasmy levels. Obs: observed; Exp: expected under random combination between heteroplasmy and TP53 genotypes (p-value= p=0.0147).

Levels of heteroplasmy			Genotype				Total
	RR		RP		PP		
	Obs (%)	Exp	Obs (%)	Exp	Obs (%)	Exp	
≤ 5%	127 (57.2)	116	75 (33.8)	87.2	20 (9.0)	18.8	222
> 5%	95 (46.8)	106	92 (45.3)	79.8	16 (7.9)	17.2	203
**Total**	222		167		36		425

## DISCUSSION

mtDNA integrity tends to decrease with age, as demonstrated by the accumulation of point mutations and deletions in a variety of tissues during aging in humans, monkeys, and rodents [35,36]. The accumulation of somatic mtDNA mutations has likely a causative role in the aging of the organisms, as suggested by animal models such as the mutator mice lacking the proofreading activity of polymerase gamma (polg) [5,6,37]. Therefore, it is reasonable to hypothesise that factors impinging upon the stability of mtDNA can affect the rate of aging in animals and humans.

p53 has a well-known role in inducing DNA repair, apoptosis, block of cell cycle and induction of cell senescence. More recently, other roles have been discovered for p53, regarding autophagy, glucose metabolism and miRNA maturation [37-40]. Among these new functions, a role in controlling mtDNA replication and repair has been proposed for p53 through the binding of polg [15] and TFAM [41]. As summarised above, it is known that human TP53 is characterised by a polymorphism at codon 72 that yields two isoforms of the p53 protein differing by one aminoacid at the level of the N-terminus Proline Rich Region, and that these two isoforms differ in the capability to localise within the cell. Since one of these two isoforms (p53R^72^) can efficiently localise at mitochondrial level, we wondered whether in cells bearing this isoform, the accumulation of alterations at the level of mtDNA was lower than in cells bearing the other isoform (p53P^72^), and whether this could have an *in vivo* consequence on the accumulation of mtDNA mutations. As a whole, our data suggest that this is the case. The mechanism by which this occurs seems to be an interaction of p53 with polg. We can not exclude, however, that other possible mechanisms take place simultaneously. For instance, it is reported that p53 binds also to HmtSSB and that this binding slightly increases the exonuclease activity of p53 capable to eliminate 3'- 8-oxo-dG nucleotides [42]. Also the binding to TFAM likely renders mtDNA more accessible to repair enzymes [17].

As a whole, the data reported in this paper and other present in the literature suggest that the two isoforms p53P^72^ and p53R^72^ are more prone to ensure the repair of the nuclear or the mitochondrial genome, respectively. Indeed, it has already been reported that cells that express in vitro the p53P^72^ isoform have a greater capability to repair damages of nuclear DNA and display a reduced production of micronuclei with respect to cells that express the p53R^72^ isoform [32]. Thus, it seems that the capability to localise at mitochondria can affect not only the activity of p53 as an inducer of transcription-independent apoptosis, but also as a cofactor of mtDNA repair, therefore influencing mtDNA stability and eventually mitochondrial efficiency.

To test whether these *in vitro* findings have an *in vivo* counterpart, we analysed the levels of mtDNA heteroplasmy in WBC from subjects with different genotype at TP53 codon 72. Indeed we found that subjects with p53R^72^/R^72^ genotype have a lower chance to accumulate heteroplasmy in the mtDNA control region (CR), suggesting that also *in vivo* this polymorphism has an effect on mtDNA stability. Previous studies had shown that the accumulation of somatic mutations in the mtDNA CR is genetically influenced by nuclear genome variability [43,44]. The variability of TP53 codon 72 may then be part of the genetic “machinery” affecting the accumulation of mtDNA somatic mutations.

It is to note that in previous reports, high levels of heteroplasmy in the mtDNA CR seem to be a genetic characteristic of human longevity, being present in centenarians and their offspring [43,44] as well as in 90+ sibpairs [45]. This may suggest that mtDNA heteroplasmy should not to be considered detrimental in all cases, being possible that certain types of heteroplasmy can provide the cell with some (still unknown) type of survival signalling. As an example, the substitution at position 150 of mtDNA CR found in centenarians seems to favour mtDNA replication [43]. If this is true, one may expect that TP53 codon 72 polymorphism can affect the survival at old age through the capability to control, at least in part, the level of heteroplasmy of the mtDNA CR. Previous studies from our group indicate that TP53 codon 72 polymorphism does not affect the human lifespan, when large cohorts of subjects from young people to centenarians are analysed [46]. However, when a more restricted age range is considered, it appears that 85+ subjects carrying the TP53P^72^/P^72^ genotype have a survival advantage over those carrying the TP53R^72^/R^72^ genotype [30]. We surmise that the effect of the TP53 codon 72 polymorphism on the level of mtDNA CR heteroplasmy might contribute to this survival advantage. Further investigations are needed to test whether this hypothesis is correct.

## MATERIALS AND METHODS

### Cells culture and treatment

p53 null HCT116 cells were a kind gift of Bert Vogelstein (Sidney Kimmel Comprehensive Cancer Center, Johns Hopkins University, Baltimore, MD, USA). They were cultured in McCoy's medium, pH 6.9, supplemented with 10% FCS and penicillin (100 units/mL)/streptomycin (100 mg/mL) in 5% CO_2_ humidified atmosphere at 37°C. They were collected at log phase of growth, counted and seeded in 6-wells plates at the concentration of 50,000/ml (total volume 2 ml). After 48 hours of culture, medium was changed and the cells were transiently transfected with 3 ng of pCMS-EGFP plasmid, either empty or expressing the EGFP-p53R^72^ or EGFP-p53P^72^ allele, using JetPEI transfection reagent (Polyplus transfection) according to the manufacturer instructions for additional 24 hours, after which 100 nM rotenone was added and left to stay for 24 hours unless otherwise indicated. After this time cells were collected and analysed by either confocal microscopy or flow cytometry. In set-up experiments cells were analysed for the level of apoptosis by staining with Propidium Iodide (PI) in hypotonic solution as previously described [28]. Analysis was performed in linear scale in order to detect also possible accumulation in G2 phase. No difference in terms of percentage of apoptotic cells was found between cells transfected with either EGFP-p53R^72^ or EGFP-p53P^72^ expressing plasmids; accumulation in G2 was also not observed under rotenone treatment (data not shown).

### Confocal microscopy

After treatment, cells were collected and stained by immunocytochemistry for polg or 8-hydroxy-2'-deoxyguanosine (8-oxo-dG). Briefly, cells were seeded into wells containing a sterile glass coverslip for confocal microscopy. After the treatments (see above), cells were washed with PBS+1% BSA, fixed in formaldehyde 2% in PBS for 10 minutes at RT and permeabilised with saponin 0.2% in PBS for further 10 minutes at RT. For polg detection, cells were incubated with anti-polg rabbit polyclonal antibodies (Pierce) (dilution 1:200) for 1 hour at 4°C and goat-anti-rabbit TRITC-conjugated IgG(H+L) (BioFX Laboratories) (dilution 1:400) for 1 hour at 4°C. Coverslips were then mounted onto a glass slide with mowiol, sealed and kept at 4°C in the dark until confocal analysis. For 8-oxo-dG detection, after fixation and permeabilisation, the cells were treated with 2N HCl for 30 minutes at RT, followed by pH re-equilibration with Borax solution 0.1M. Slides were then washed with PBS+1% BSA and incubated with anti-8-oxo-dG mouse monoclonal antibodies (Trevigen) (dilution 1:1000) for 4 hours at 4°C and goat-anti-mouse RPE-conjugated monoclonal antibody (Dako) (dilution 1:250) for 1 hour at 4°C. In some experiments, bis-benzimide (Hoechst 33258) 250ng/ml was added to counterstain nuclei. Slides were analysed with a “Leica DMIRE2” inverted research microscope connected with a TCS SP2-AOBS system (Leica, Germany) equipped with blue COH diode (405nm/25mW), Ar (458nm/5mW) (476nm/5mW) (488nm/20mW) (496nm/5mW) (514nm/20mW), HeNe (543nm/1.2mW), HeNe (594nm) (Orange) and HeNe (633nm/102mW) lasers. The microscope and the AOBS system were controlled by the LCS software (Leica). The slides were observed under a HCX PL APO 40x/1.25 – Oil- objective. In order to avoid overlaps between fluorochrome excitations/emissions during scanning procedures, images where acquired in separate channels for each fluorochrome. Merged figures derived from the software-assisted integration of horizontal xyλ-sections (line average= 2; number of scansions per line= 3). Colocalisation of different fluorochromes was ascertained using the measuring and analysis functions of LCS software Version 2.0, 2001 (Leica, Germany).

### Flow cytometry

Cytofluorimetric analyses were performed using a FACScalibur cytometer (BD), equipped with an argon laser on a minimum of 10,000 cells per sample, acquired in list mode. Briefly, cells were detached from substrate with 1% trypsin in PBS for 2 minutes and treated as described for 8-oxo-dG detection. Analysis of 8-oxo-dG specific fluorescence was performed on cells electronically gated for being positive for EGFP fluorescence (meaning that they are expressing p53) and compared to those negative for EGFP (meaning that they are not expressing p53) in the same sample, thus avoiding experimental variability. The experiments were repeated three times. Mitochondrial Membrane Potential (MMP) was assessed by using the potentiometric dye Tetramethyl-rhodamine methyl esther, perchlorate (TMRM, Molecular Probes). Staining with 150 nM TMRM for 15 min at room temperature was followed by washing in PBS and analysis. Multiparametric analysis allowed for the simultaneous detection of TMRM (red) and EGFP (green) fluorescences.

### Generation of stable transfectant

In order to obtain a higher percentage of transfected cells, we generated stably transfected cell lines expressing the two p53 isoforms using PC3 p53 null cells. An HA-epitope tag was added to the N-terminal position of p53 cDNA sequence of both codon 72 Arginine and Proline isoforms using PCR with the following primers: Forward, p53 NotI HA tag 5'- GCG GCC GCG GCG CGC CCG GGA TCC TGA TCA GCA GGC GCC ATG TAC CCA TAT GAT GTT CCA GAT TAC GCT GAG GAG CCG CAG TCA GAT CCT -3'; Reverse, p53 BglII site 5'- GCA GAT CTA TTT AAT TCA GTC TGA GTC AGG CCC TTC T -3'. The restriction sites NotI and BglII were used to ligate the PCR product into the retrovector LNXCO3 that supports IPTG inducible expression.

PC3 subline 3'SS6 was derived after transfection with the murine ecotropic retrovirus receptor and LacI repressor genes as previously described [47]. The LNp53CO3 vector for both isoforms was constructed by cloning the entire coding sequence of the human p53 cDNA in the NotI and BglII sites of IPTG-inducible retroviral vector LNXCO3 [47]. Insert-free vector LNXCO3 was used for control infections. Both isoforms of LNp53CO3 and LNXCO3-transduced cell populations were selected with 600ug/ml G418. Cell lines were cloned by plating 500-2000 cells per 150 mm tissue culture plate and individual colonies collected. Expression in PC3 cells was induced with 100 μM IPTG for two weeks and clones were selected for 100% expression, evaluated by flow cytometry (Cytomics ™ FC 500, Beckman Coulter Inc. Brea, Ca) using an antibody to the HA-epitope (Covance Inc., Emeryville, Ca). For flow cytometry, PC3 cell lines expressing both isoforms of p53 were fixed with 70% ethanol and permeabilised with PBS containing 0.2% Triton X-100. Staining was performed using 1:1000 HA-epitope antibody and a fluorescent secondary antibody.

To confirm that the cells retained the correct isoform of p53, they were analysed by retrotranscription and amplification of the gene introduced with the viral vector, and digestion with BstUI restriction enzyme. PCR yielded a product of about 1,252 bp, as expected, and digestion with BstUI produced three fragments (722, 284 and 252 bp) from cells transfected with the R isoform, and two fragments (722 and 536 bp) from those transfected with the P isoform (data not shown), confirming the attribution of the isoform expressed by the cells.

### Immunoprecipitation and immunoblotting

p53 expression in PC3 cells was induced with 100 μM IPTG. Subconfluent cells were treated with 10 nM rotenone (Sigma-Aldrich, Inc. St. Louis, MO) for 72 h. Rotenone treated and untreated cells were extracted with Lysis Buffer II (Pepscan Presto, Lelystad, The Netherlands), sonicated and centrifuged at 4000 × g before immunoprecipitation. To immunoprecipitate DNA polymerase gamma, 1000 μg of cell extract was incubated with 1.6 μg of a rabbit polyclonal antibody (Pierce PA121791, *Pierce* Rockford, IL, USA) for 5 hours at 4^o^ C. The immune complexes were captured with protein A/G sepharose. The immunoprecipitates were analysed by western blot using an antibody to the HA-epitope tag on both p53 isoforms.

### mtDNA integrity assessment

#### mtDNA copy number

The mtDNA content was measured by real-time PCR on a mtDNA encoded gene rarely deleted (ND1) normalised by simultaneous measurement of nuclear DNA encoded β actin gene. The primers sequences are as follow: ND1: L3485-ND1_Forward: CCC TAA AAC CCG CCA CAT CT; H3532-ND1_Reverse: GAG CGA TGG TGA GAG CTA AGG T. β Actin: Forward: ACC CAC ACT GTG CCC ATC TAC; Reverse: TCG GTG AGG ATC TTC ATG AGG TA. The quantitative PCR was conducted on a Rotor gene Q 6000 system (Qiagen) in a 10 μl reaction in different tubes containing 0.3μM each of the forward and reverse primer (ND1 and β actin genes), 15 ng of DNA sample and 1X Mesagreen reaction mix (Eurogentec). The PCR condition was set as follow: 95°C for 5 min, 40 cycles at 95°C 15 s and 60 °C 1 min. The Ct Values for both the genes were determined. Each measurement was carried out in duplicate and repeated three times, the same calibrator sample was used in each run. The analysis was performed normalising against the calibrator and the result was the average of the three repeated experiments.

#### Nested PCR

A nested PCR analysis was performed to screen for low levels of the 4,977 bp deletion in mtDNA. Primers and PCR conditions are previously reported [34], briefly the PCR condition was: pre-denaturation at 94°C for 5 min, then 30 cycles at 94°C for 10s, 58°C for 45 s and 72°C for 50 s; and a final extension at 72°C for 10 min. PCR products were visualised in a 2% agarose gel. The presence of the 4977-bp deletion was indicated by the appearance of a 358 bp band, which was verified by the presence of a positive control sample harbouring the 4,977 bp deletion.

#### Long PCR

The 11.3 kb long-PCR to amplify almost full-length of mtDNA was carried out to detect mtDNA multiple deletions using forward primer corresponding to L-strand nt-3485 to 3519, backward primer H-strand nt-14820 to 14786 and Takara LA Taq DNA polymerase (Takara Shuzo Corp., Japan) as previously reported [48]. Briefly the PCR condition was: pre-denaturation at 94°C for 3 min, then 30 cycles at 98°C for 10s and 68°C for 15 min followed by a final extension at 72°C for 12 min. PCR products were visualized in a 1% agarose gel. The 11.3-Kb band represents the wild type mtDNA, all the other bands indicate the deletions.

### *In vivo* correlation between mtDNA heteroplasmy and TP53 codon 72 genotype

#### Sample

A total of 425 unrelated subjects (191 men and 234 women, age range 60-108 years; median ages 83.09 (±15.04) and 86.06 (±13.29) years, respectively) were recruited to participate in this study. All the subjects were born in Calabria (Southern Italy) and their ancestry in the region has been ascertained up to the grandparents' generation. The sample has been collected in the frame of several recruitment campaigns carried out for monitoring the quality of aging in Calabria from 2002. Younger subjects were contacted through general physicians. Subjects older than 90 years were identified through the population registers and then contacted by specialised personnel and invited to join the study. Each subject was submitted to a home-based interview by a trained operator, with the administration of a structured questionnaire, validated at European level. The questionnaire was aimed to the collection of socio-demographic information, evaluation of physical, cognitive, depressive status, sensory deficits, medications, and self-reported health status. In addition, common clinical haematological tests were performed. Subjects with dementia and/or neurologic disorders were not included. All the subjects had given informed consent for studies on aging carried out by our research group. White blood cells (WBC) from blood buffy coats were used as source of DNA.

#### p53 codon 72 genotype determination

DNA was extracted using phenol/chloroform, according to standard procedures. TP53 genotyping was performed as follows: a 156bp sequence containing the polymorphic site was amplified by PCR using forward 5' GACCCAGGTCCAGATGAAGCT -3' and reverse 5'-ACCTACCAGGGCAGCTACGGT-3′ primers. Reactions were done in a 50-μL mixture containing DNA aliquot (100 ng), 1× PCR buffer, 0.2μM of each primers, 0.2mM of each dNTP, 1.5mM MgCl_2_, and 1 U GoTaq DNA polymerase (Promega). The PCR conditions were set as initial denaturation at 96°C for 3 minutes followed by 30 cycles containing 94°C at 30 seconds, 60°C for 30 seconds, and 72°C for 1 minute. The amplicon was digested with 3U of *Bst*UI (Promega) for 4 h at 60°C and the products were analysed on 2% agarose gel.

#### Quantification of the heteroplasmy by DHPLC

A 300bp fragment of the mitochondrial DNA control region (nt 16531-261; 300bp) was PCR amplified from each DNA sample and submitted to DHPLC as previously described in detail [44].

#### Statistics

The goodness-of-fit to the Hardy-Weinberg equilibrium was calculated by the chi-square test. Statistical comparisons by Pearson Chi-Square and univariate logistic analysis were performed on SPSS 15.0 (SPSS Inc., Chicago, IL). *p* values less than 0.05 were considered statistically significant.

## Abbreviations

ROS: reactive oxygen species
WBC: white blood cells
Polg: polymerase gamma
8-oxo-dG: 8-hydroxy-2'-deoxyguanosine
mtDNA: mitochondrial DNA
mtDNA CR: mitochondrial DNA control region
